# Multidecadal preindustrial methane variability can be explained by noise in the source–sink imbalance

**DOI:** 10.1073/pnas.2601235123

**Published:** 2026-06-15

**Authors:** Eric J. Mei, Gregory J. Hakim, Cristian Proistosescu, Thomas K. Bauska, Christo Buizert, Alexander J. Turner

**Affiliations:** ^a^ Department of Atmospheric and Climate Science, University of Washington, Seattle, WA 98195; ^b^ Department of Climate, Meteorology, and Atmospheric Sciences, University of Illinois at Urbana Champaign, Urbana, IL 61820; ^c^ Department of Earth Sciences and Environmental Change, University of Illinois at Urbana Champaign, Urbana, IL 61820; ^d^ British Antarctic Survey, Cambridge CB3 0ET, United Kingdom; ^e^ College of Earth, Ocean, and Atmospheric Sciences, Oregon State University, Corvallis, OR 97331

**Keywords:** methane, ice core, internal variability

## Abstract

Ice core records of preindustrial methane indicate variability of ±30 ppb (±5%) on multidecadal-to-centennial timescales. Previous work has attributed these excursions to low-frequency or episodic changes in methane sources due to large-scale climate variability (e.g., the Little Ice Age) or human activity. Here, we explore what source and sink dynamics are consistent with ice core variability and show that the preserved variability can arise solely from white noise fluctuations in the source–sink imbalance. A simple model driven by unforced random perturbations, when integrated by the methane lifetime and firn processes, produces synthetic ice core records that match the spectral features of observations. Thus, fast-varying sources or sinks previously assumed too transient to affect ice core variability, such as weather-driven fluctuations in methane oxidants and emissions (e.g., interannual wetland variability), can produce the observed variability. Given large structural uncertainty in methane budget dynamics, we expand our model to quantify how variability in atmospheric methane and its drivers changes with the assumed dominant timescale of source–sink imbalance variability. Our results show that any source–sink imbalance timescale shorter than a century is consistent with the ice core record. Shorter timescales imply greater atmospheric variability but require larger-amplitude source–sink imbalance fluctuations. A key consequence is that rapid preindustrial source–sink variability (timescales ≲ 1 y) could explain modern methane growth rate variability. Constraining methane source and sink dynamics with preindustrial ice core records could therefore provide more robust priors for evaluating modern methane trends.

## Significance

Prior to the Industrial Revolution, ice core methane records show multidecadal-to-centennial variability usually attributed to slow climate changes or human activity. We show that this variability can instead arise from fast, random fluctuations in methane’s sources and sinks once the smoothing effects of methane’s atmospheric lifetime and firn processes are accounted for. This result shows that ice core methane excursions do not require slow or synchronous changes but can arise from fast processes such as variability in oxidants and year-to-year changes in wetland and biomass burning emissions. If fast source and sink variability dominates the methane budget, natural variability in preindustrial atmospheric methane is comparable to modern year-to-year variability.

Atmospheric methane is the second-most important anthropogenic greenhouse gas after carbon dioxide. It has risen sharply since the onset of the Industrial Era to concentrations unprecedented in the observational record (1). Yet present trends show large interannual to decadal variability, including the “methane stabilization” in the early 2000s during which methane concentrations remained steady before rising again (2). Identifying the causes of this variability is difficult due to limited measurements and poor understanding of processes that govern the global methane budget (1, 3, 4). In particular, competing hypotheses for methane variability often disagree on the contribution of natural processes to observed present trends (5–17), complicating attribution to anthropogenic activity.

**Fig. 1.**
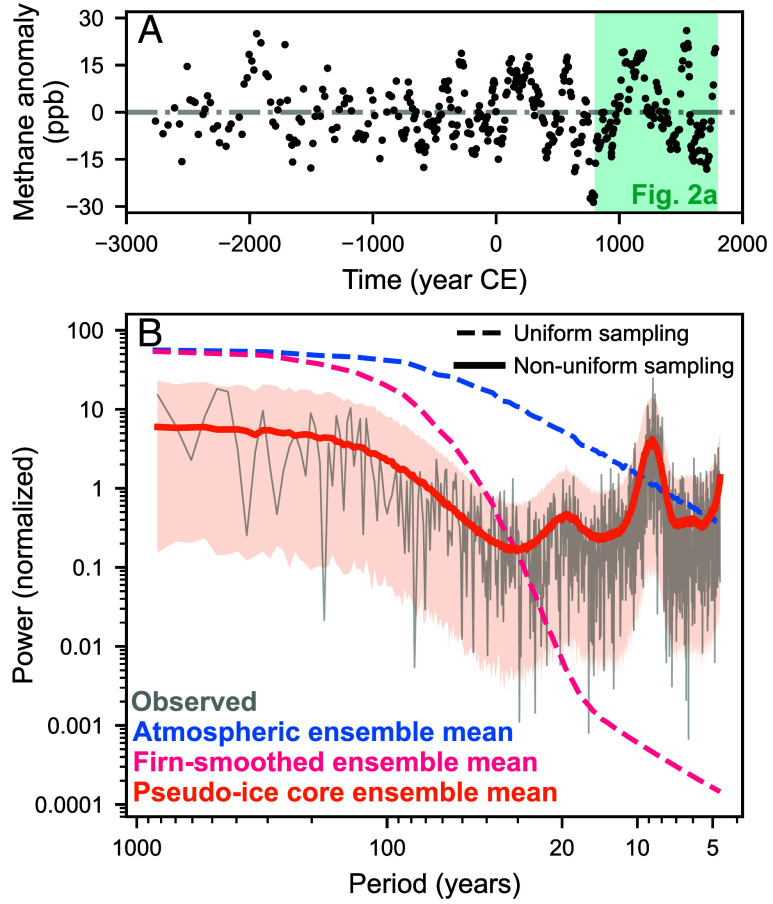
Comparison of power spectral density of ice core methane observations and simulations from Eq. **1**. (*A*) Ice core methane observations from WAIS Divide (18) detrended with a second-order polynomial fit (*SI Appendix*, Fig. S1). Teal shading encompasses the subset of data shown in Fig. 2*A*. (*B*) Lomb–Scargle (19) power spectral density of ice core methane observations (gray) and simulations of atmospheric methane (blue), firn-smoothed methane (magenta), and pseudo-ice core methane observations (orange). Dashed lines show ensemble means of 1,000-member ensembles sampled uniformly in time (atmospheric and firn-smoothed signals), and the solid line shows the ensemble mean of pseudo-ice core simulations sampled at the observed times. The pseudo-ice core signal and the observations are normalized to unit variance; the atmospheric and firn-smoothed spectra are scaled by the same normalization factor to maintain variance consistency across all signals. Shading indicates the 95% CI of pseudo-ice core ensemble.

Measurements of methane trapped in polar ice cores provide a long-term record for understanding natural methane variability in the absence of anthropogenic influence. Submillennial variability in preindustrial ice core methane is characterized by excursions of ±30 ppb (±5%) on multidecadal-to-centennial timescales (Fig. 1*A*) and is seen across both hemispheres (18, 20–25). Such variability is driven by an imbalance between methane sources and sinks. Preindustrial sources of methane include wetlands, biomass burning, human agriculture, and minor geological sources (26–29). Sinks are dominated by chemical oxidation with hydroxyl radicals (OH) in the troposphere, with minor contributions from oxidation by other radicals and from soil uptake (26, 29).

Prior studies of preindustrial methane variability often use box model representations of the atmosphere to simulate ice core variability, in which methane oxidation rates and meridional transport are prescribed (e.g., refs. 18, 20, 22, and 23). Forward model simulations or statistical inversions are conducted such that the variations in methane sources satisfy the observed methane record. These studies often attribute multidecadal-to-centennial methane excursions in the ice core record to synchronous variability in the methane budget. As such, methane anomalies in the record have been attributed to slow, large-scale changes in climate that affect wetland or biomass burning emissions (18, 20, 22–24, 30) or to contemporaneous events in human history, such as disease outbreaks, war, and colonization that impact agriculture or land use (18, 22, 31). The assumption that source–sink imbalance variability is multidecadal-to-centennial has ruled out the methane sink as a significant contributor to ice core variability in these studies as chemical oxidant variability is assumed to be high-frequency (e.g., refs. 18, 22, and 24).

Here, we use a simple model of atmospheric methane variability to demonstrate that the preindustrial methane budget need not vary on slow, multidecadal-to-centennial timescales to be consistent with the ice core record. Instead, a null hypothesis of white noise variability in methane’s sources and sinks can explain observed ice core methane variability. Given the large structural uncertainty in source and sink dynamics, we use our model to explore the range of timescales and magnitudes of methane budget variability that are consistent with the ice core record. This work outlines the potential frequency and magnitude of unforced variability in atmospheric methane and its sources and sinks.

## Can White Noise Cause Multidecadal-to- Centennial Methane Variability?

Atmospheric methane variability is driven by an imbalance of the sources and sinks of methane. Let C be the global atmospheric methane burden. Its continuity equation is $dC/dt=S-\tau^{-1}C$, in which sources of methane are represented by S. Methane loss is first order with respect to a steady-state atmospheric lifetime (τ), reflecting the dominant methane loss process of chemical oxidation. We investigate submillennial perturbations about a steady-state concentration of methane ($\bar{C}=\bar{S}\;\bar{\tau }$) by linearizing the continuity equation (see *Materials and Methods* for full discussion of this derivation):

[1] $$\begin{array}{c} \frac{dC^{\prime }}{\mathrm{dt}}=-\tau_{C^{\prime }}^{-1}C^{\prime }+\varepsilon . \end{array}$$

$C^{\prime }$ is the anomaly of methane about its mean ($C^{\prime }=C-\bar{C}$). Linearizing the loss rate ($\tau^{-1}C$) yields a methane “perturbation lifetime” ($\tau_{C^{\prime }}$), the effective e-folding timescale governing how quickly a methane anomaly relaxes back toward the mean once chemical feedbacks are included (32). This perturbation lifetime is estimated to be between 8 to 12 y by models but is uncertain (33, 34). We use a preindustrial perturbation lifetime of 10 y for this work (34), and our conclusions are not sensitive to this choice (*SI Appendix*, Fig. S3). ε represents anomalies in the balance of methane’s sources and sinks (“source–sink imbalance”) that perturb atmospheric methane concentrations. Source–sink imbalance perturbations could arise from external forcings or natural internal variability, such as interannual changes in wetland methane emissions or OH concentrations.

To test prior assumptions that multidecadal-to-centennial scale changes in the ice core record must be due to slowly varying sources, we assume that the dynamics of the methane source–sink imbalance (ε) can instead be approximated with a Gaussian white noise process. White noise represents a methane budget dominated by weather-timescale fluctuations such as biomass burning events or OH variability. Such variations in OH could be caused by day-to-day changes in ozone photolysis frequency, specific humidity, or natural emissions of reactive nitrogen and carbon (35). This white noise approximation comprises a null hypothesis for the temporal behavior of methane budget dynamics: Because the white noise process is uncorrelated in time, it has no memory and has equal power at all frequencies. We simulate 1,000 realizations of Eq. **1** matched to the duration of the West Antarctic Ice Sheet (WAIS) Divide WDC06A ice core record (18) to compare the spectral features of our simple model to observations (Fig. 1). It is important to note that the intensity of the white noise in the source–sink imbalance is tuned such that simulated pseudo-ice core variance matches the variance of the WAIS Divide record. Our conclusions are insensitive to the record used (*SI Appendix*, Figs. S1 and S2).

Fig. 1*B* shows that methane anomalies from Eq. **1** (dashed blue line) suppress the high-frequency power of the input white noise source–sink imbalance to produce a smoothed signal. This result is consistent with those of canonical stochastic climate models, in which reservoirs with memory integrate high-frequency variability and respond slowly (i.e., “Hasselmann models;” 36–38). Here, the reservoir with memory is the atmospheric chemical system that damps methane budget perturbations on a decadal timescale ($\tau_{C^{\prime }}=10$ y).

Spectra of atmospheric methane from Eq. **1** are not directly comparable to the ice core records without smoothing of the signal from firn densification. As atmospheric methane is trapped in polar ice, processes in the firn column including turbulent mixing, molecular diffusion, and bubble close-off (39–41) integrate the atmospheric signal on decadal timescales before it is archived in ice cores. This smoothing further suppresses high-frequency variability (dashed magenta line in Fig. 1*B*).

Sampling the firn-smoothed signal at the same irregular intervals as the measurements in the WAIS Divide record (Fig. 1*A*) yields spectra (solid orange line) that capture the observed continuum from low- to high-frequency variability as well as sampling artifacts that most prevalently appear in the peaks near 20-, 10-, and 5-y periods. Nonuniform sampling also leaks low-frequency power into higher frequencies, which distorts the power spectra at all frequencies. These artifacts combined with uncertainties in the gas age of the measurements limit the ability of the power spectra to precisely constrain the timescales of the methane system. Artifacts from nonuniform sampling in time occur in all records investigated (see *SI Appendix*, section 2 for a detailed discussion of irregular sampling). To reiterate, the most prominent features in the power spectra shown in Fig. 1*B* at 10 and 20 y are entirely due to the irregular sampling of the ice core. Although shown here for methane, this finding also has implications for the interpretation of ice core records of other species.

These results suggest a null hypothesis of white noise in the methane source–sink imbalance, integrated by the methane lifetime and firn processes, can explain the observed multidecadal-to-centennial variability in ice core methane observations—provided the source–sink imbalance amplitude is sufficiently large. We set this amplitude by matching the observed ice core variance, which we later use to constrain source–sink dynamics. This parsimonious explanation requires only two parameters ($\tau_{C^{\prime }}$ and ε), far fewer than many prior reconstructions, and this phenomenon is common in geophysical processes, in which many signals are slow responses to fast stochastic variability (38). In this sense, multidecadal-to-centennial-scale excursions in the ice core methane record do not intrinsically require large-scale synchronous variability in methane’s sources or sinks (e.g., low-frequency climate variability or anthropogenic forcing). This motivates the next question: *what source–sink dynamics are consistent with ice core methane variability?*

## What Source–Sink Dynamics Explain Methane Variability?

Though a white noise assumption for the methane source–sink imbalance can explain observed ice core variability, it is unclear whether this assumption is physically appropriate as true sources and sinks may have memory. In physical stochastic climate models, white noise processes that approximate fast-timescale dynamics (e.g., weather) may be validated by observations (e.g., ref. 37) or model simulations. Even for present trends, the dynamics of sources and sinks that govern the global methane budget are poorly understood (4). This is exacerbated in the preindustrial. These uncertain methane source–sink dynamics obscure the true frequency and magnitude of unforced variability.

While the white noise null hypothesis provides a statistical baseline for methane variability, real unforced source–sink processes may operate on characteristic timescales that deviate substantially from white noise. To assess how changes in such dynamics might modify unforced variability, we expand beyond our white noise null hypothesis. Here, we investigate characteristics of source–sink dynamics that could drive ice core methane variability.

We now relax the white noise assumption for ε in Eq. **1** with a simple model for source–sink imbalance dynamics that allows for memory:

[2] $$\begin{array}{c} \frac{d\varepsilon }{\mathrm{dt}}=-\tau_{\varepsilon }^{-1}\varepsilon +\eta . \end{array}$$

**Fig. 2.**
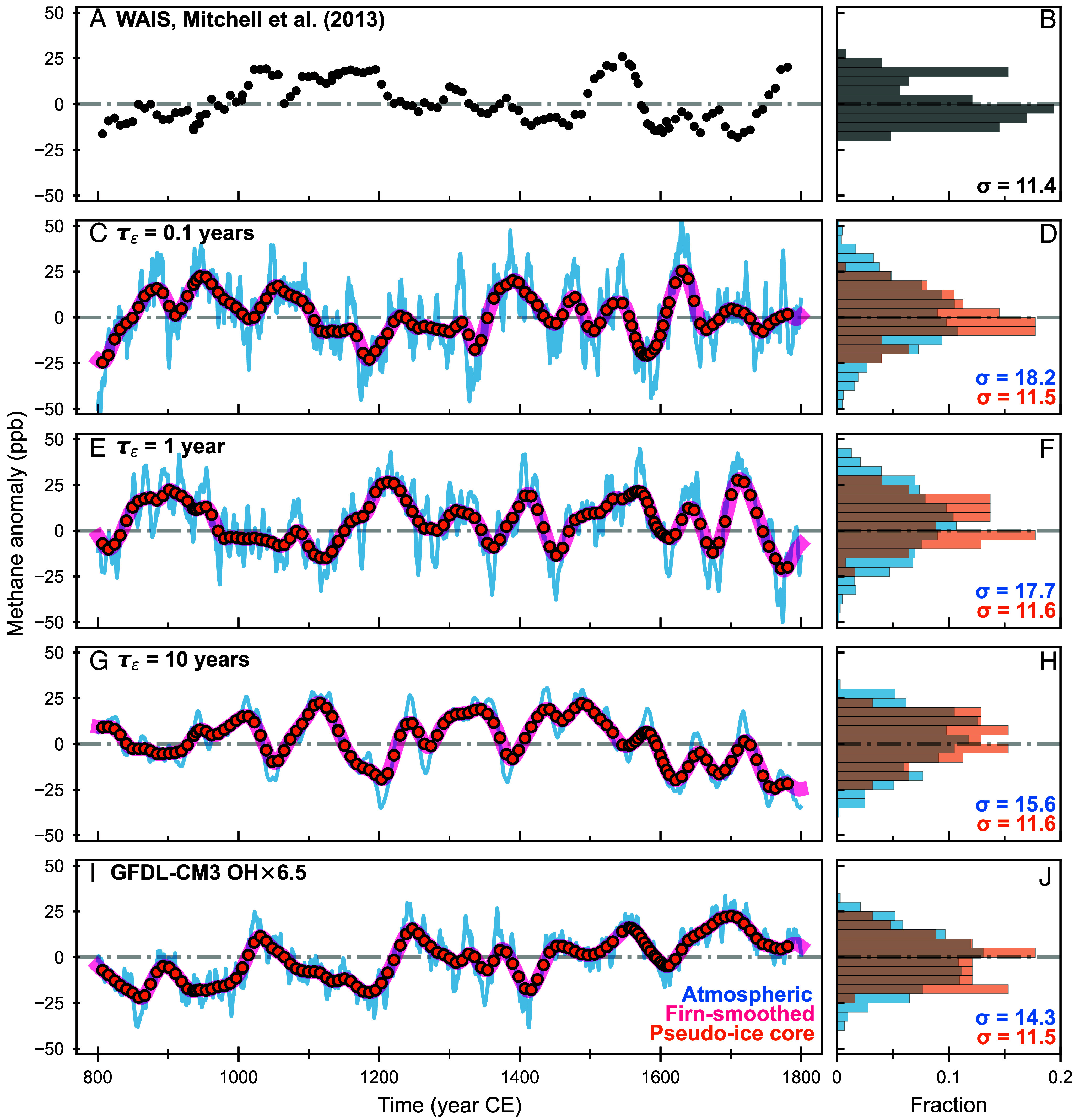
Comparison of ice core methane observations to simulated pseudo-ice cores with different source–sink dynamics. (*A*) 1,000-y subset of ice core methane observations from WAIS Divide shown in Fig. 1*A*. (*B*) Histogram of observations shown in (*A*), along with the SD of the distribution. (*C*–*H*) Realizations of Eqs. **1** and **2** for different timescales of the source–sink imbalance ($\tau_{\varepsilon }$, Eq. **2**) as indicated by panel labels. Simulations of atmospheric methane (blue), firn-smoothed methane (magenta), and pseudo-ice core observations sampled at the same time intervals as the WAIS Divide observations (orange). Atmospheric and firn-smoothed methane concentrations are taken at 1-y intervals. Timeseries are shown in panels (*C*, *E*, and *G*), while corresponding histograms of simulated atmospheric methane (blue) and pseudo-ice core observations (orange) are shown in panels (*D*, *F*, and *H*), respectively, along with the SDs of the distributions. (*I*) Simulation of atmospheric, firn-smoothed, and pseudo-ice core methane performed with the source–sink imbalance prescribed as a 1,000-y integration of OH from the GFDL-CM3 model (42, 43) scaled by a factor of 6.5. The timestamps of the simulation are recentered to match the observed methane record. (*J*) Histograms of simulated atmospheric methane and pseudo-ice core observations shown in (*I*), along with the SDs of the distributions.

We assume that source–sink imbalances can be characterized by a single dominant e-folding decorrelation timescale ($\tau_{\varepsilon }$) and variance ($\sigma_{\varepsilon }^{2}$), which is controlled by the intensity of the Gaussian white noise perturbations (η) to the source–sink imbalance. In the limit where $\tau_{\varepsilon }$ is very short relative to the methane lifetime, we recover the original white noise null hypothesis. We conduct sensitivity tests across a range of parameters ($\tau_{C^{\prime }}$, $\tau_{\varepsilon }$, $\sigma_{\varepsilon }^{2}$) to assess which timescales and magnitudes of source–sink variability can reproduce the observed ice core variability. Fig. 2 shows realizations of Eqs. **1** and **2** using a 10-y methane perturbation lifetime and source–sink imbalance timescales of $\tau_{\varepsilon }$ = 0.1, 1, and 10 y. These order-of-magnitude changes in timescale approximate source–sink imbalances dominated by high-frequency variability (e.g., variability in oxidant concentrations), interannual variability (e.g., wetland emissions responding to internal climate variability), and low-frequency variability (e.g., changes in wetland extent), respectively.

Though simulated variability in atmospheric methane is strikingly different among the range of source–sink imbalance timescales (blue lines in Fig. 2), smoothing of the atmospheric signal by the firn model results in pseudo-ice core observations (orange points) that are statistically similar to true observations for all e-folding timescales. We simulate periods of both positive and negative anomalies in the pseudo-ice core record that persist for decades to centuries, consistent with the actual ice core record from WAIS Divide. Similarly, we simulate large and fast changes in pseudo-ice core record without the need for external forcing (e.g., ∼50 ppb increase over ∼40 y near year 1600 in Fig. 2*C*). Source–sink imbalance timescales both shorter and longer than those shown, up to around 100-y timescales, produce pseudo-ice core records with similar characteristics (*SI Appendix*, Fig. S6).

In light of this finding, we revisit previous assumptions that high-frequency variability in methane’s chemical sinks (i.e., OH) could not have caused significant variability in ice core observations. Fig. 2*I* shows a simulation of Eq. **1** with a source–sink imbalance using constant methane sources and a sink prescribed by OH variability from a preindustrial control simulation of the Geophysical Fluid Dynamics Laboratory Coupled Model 3 (GFDL-CM3) (42, 43). Both the simulated atmospheric methane and pseudo-ice core record show multidecadal-to-centennial variability that is consistent with the true ice core record.^*^ Therefore, it is plausible that the true variability in methane’s sinks could significantly affect interpretation of ice core methane variability. This is consistent with prior work suggesting that interannual variability in OH can influence inferred methane emissions and variability (8, 9, 45, 46).

Though many source–sink imbalance timescales result in similar pseudo-ice core observation statistics, the characteristics of atmospheric methane variability produced by these timescales differ substantially. As the timescale of the source–sink imbalance decreases, the range and frequency of the atmospheric methane variability increases (Fig. 2*C*–*H*). Shorter timescales of the source–sink imbalance decrease the autocorrelation of atmospheric methane and increase its high-frequency power, which is efficiently filtered by decadal smoothing from the firn processes. At short timescales ($\tau_{\varepsilon }≲1$ y), firn smoothing obscures frequent and substantial atmospheric methane excursions. Atmospheric concentrations often change by 25 to 50 ppb over one to two decades with little corresponding change in pseudo-ice core observations (Fig. 2 *C* and *E*).

**Fig. 3.**
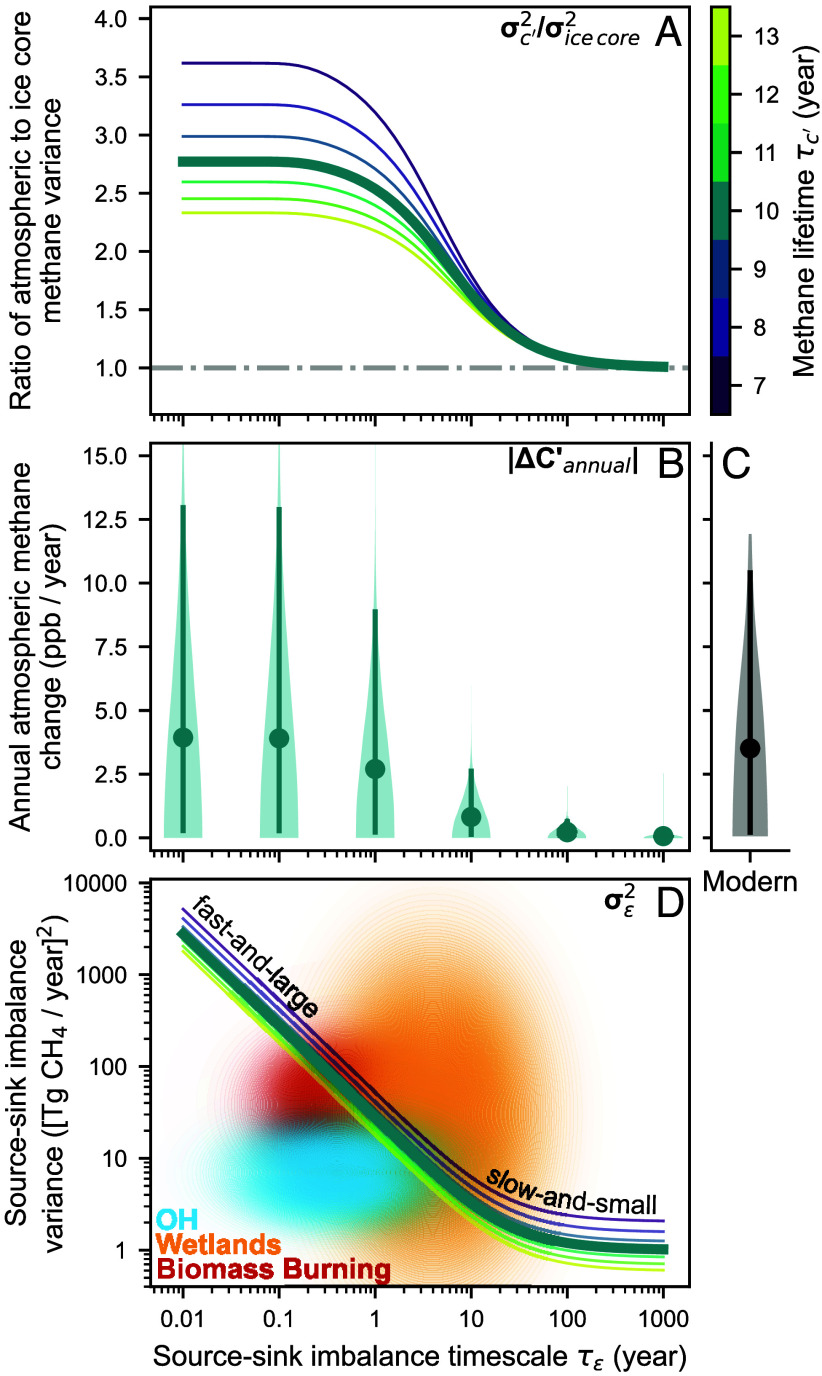
Variance required in atmospheric methane and the associated source–sink imbalance to produce the ice core methane variance observed in the WAIS Divide record over a range of source–sink imbalance timescales ($\tau_{\varepsilon }$). (*A*) Variance inflation ratio of atmospheric methane to ice core methane caused by smoothing from the methane lifetime and the WAIS Divide firn. The horizontal dashed line indicates a ratio of 1. (*B*) Distribution of the absolute change in annual-average atmospheric methane for $\tau_{C^{\prime }}=10$ y. Violin plots show distributions of 1,000,000 simulated years for each timescale with atmospheric variances consistent with panel (*A*). The vertical bar shows the central 95% quantile range of the distributions, and the point shows the median. (*C*) Violin plot for the same distribution as (*B*), but for modern measurements of methane (47) assuming a linear anthropogenic forced trend (n=40). Again, the vertical bar and point show the 95% quantile range and median of the distribution. (*D*) Mean variance of the source–sink imbalance required to produce ice core methane variance. Two regimes of source–sink imbalance are labeled: “fast-and-large” and “slow-and-small.” Shaded patches indicate possible parameter spaces for OH oxidation (cyan), wetland methane emissions (orange), and biomass burning methane emissions (red) (*SI Appendix*, section 3). (*A* and *D*) Solutions shown for $\tau_{C^{\prime }}$ from 7 to 13 y. The thick line is the solution for $\tau_{C^{\prime }}=10$ y.

To quantify how much more variability is present in the atmospheric signal than the archived ice core signal, we derive the expected ratio of atmospheric methane variance ($\sigma_{C^{\prime }}^{2}$) to ice core methane variance ($\sigma_{\mathrm{ice}\;\mathrm{core}}^{2}$) from Eqs. **1** and **2**, and the firn smoothing filter (Fig. 3*A*; Eq. **13** in *Materials and Methods*, derivation is shown in *SI Appendix*, section 1). This “variance inflation ratio” increases with shorter source–sink imbalance timescales. Thus, the ice core record preserves less variance than the atmosphere as sources or sinks become higher-frequency, which is evident in Fig. 2*D*, *F*, and *H*. Interestingly, this variance inflation ratio plateaus for timescales shorter than 0.1 y or longer than 100 y. At timescales longer than 100 y, the atmospheric variance converges to ice core variance because the source–sink imbalance lacks high-frequency power. At timescales shorter than 0.1 y, high-frequency power in the source–sink imbalance is indistinguishable from white noise from the perspective of the methane lifetime’s decadal memory (*SI Appendix*, Fig. S8). Further increases in high-frequency source–sink power are not transferred to the atmospheric signal. In other words, we reach the “white noise limit” at $\tau_{\varepsilon }\leq 0.1$ y, and the variance inflation ratio converges to ∼2.8. We note that these plateaus are relative to the methane lifetime. For example, the variance inflation ratio at this limit increases at shorter methane lifetimes due to increased high-frequency atmospheric methane variability. Conversely, longer methane lifetimes decrease the ratio. Inflation of atmospheric variance at subcentennial source–sink timescales indicates that reconstructions of past atmospheric methane could require substantially more variability than observed in ice core records. This behavior has a strong dependence on the uncertain timescales ($\tau_{C^{\prime }}$, $\tau_{\varepsilon }$) of the methane system.

The possibility of large, high-frequency preindustrial atmo- spheric methane variability has important implications for modern interannual methane variability. Here we pose the question, *“is preindustrial methane variability large enough to explain variability in modern methane growth rates?”* We simulate a 1,000-member ensemble of Eqs. **1** and **2** across a range of source–sink imbalance timescales to calculate the distribution of annual mean methane concentration changes (Fig. 3*B*). Source–sink imbalance timescales that are sufficiently short (i.e., $\tau_{\varepsilon }≲1$ y) capture the range of variability observed in modern methane growth rates (Fig. 3*C*; ref. 47), assuming a linear forced anthropogenic trend (*SI Appendix*, Fig. S7). Thus, preindustrial natural variability could be sufficient to explain modern interannual methane variability. The presence of large natural variability would accommodate a wide range of proposed drivers of modern methane growth rate variability (5–17), including variability in oxidants and emissions, and suggests that attribution of interannual methane variability to anthropogenic forcing may be difficult. Constraints on the dominant timescales of preindustrial methane source and sink dynamics could provide direct empirical evidence for how much modern variability should be expected from internal variability alone.

### Constraints on Methane Source–Sink Dynamics.

As mentioned earlier, we scale the amplitude of the source–sink imbalance such that the pseudo-ice core record matches the observed ice core variance. Qualitatively, processes with short timescales require a large source–sink imbalance amplitude and, conversely, processes with long timescales require a small source–sink imbalance amplitude. We therefore explore the range of source–sink imbalance amplitudes (i.e., $\sigma_{\varepsilon }^{2}$) that can reproduce the observed ice core variability as a function of timescale ($\tau_{\varepsilon }$). Exploration of this parameter space may provide constraints on the methane source–sink dynamics that are consistent with the observational constraints.

Fig. 3*D* shows the emergence of two regimes of source–sink imbalance dynamics: a “fast-and-large” regime and a “slow-and-small” regime. Eq. **12** in *Materials and Methods* and the preceding equations show the derivation of the relationship between $\sigma_{C^{\prime }}^{2}$, $\sigma_{\varepsilon }^{2}$, and $\tau_{\varepsilon }$. In the fast-and-large regime ($\tau_{\varepsilon }$ is small), the source–sink imbalance provides variance primarily at high frequencies, which are strongly damped by the methane lifetime and further filtered by firn smoothing. To match the observed ice core variance after this filtering, the required source–sink variance ($\sigma_{\varepsilon }^{2}$) must therefore increase as $\tau_{\varepsilon }$ decreases. Beyond the “white noise limit” ($\tau_{\varepsilon }\leq 0.1$ y in Fig. 3*A*), the atmospheric response no longer changes, but the required source–sink variance continues to increase because an increasing share of variance is filtered out before being archived (*SI Appendix*, Fig. S8). This provides a feasibility constraint on very short $\tau_{\varepsilon }$.

In the slow-and-small regime ($\tau_{\varepsilon }$ is large), the source–sink variability is largely low-frequency and survives smoothing, so the required source–sink variance converges to $\sigma_{C^{\prime }}^{2}/\tau_{C^{\prime }}^{2}$. However, at very long timescales ($\tau_{\varepsilon }>100$ y), pseudo-ice core records no longer resemble the observed record (*SI Appendix*, Fig. S6). This does not rule out slower sources or sinks as major contributors to ice core variability, but it suggests that matching the record requires at least one shorter-timescale source–sink component.

As the methane budget is comprised of multiple sources and sinks with different dynamics, these regimes outline dynamical characteristics required to explain ice core variability. Sources or sinks that fall far below the required variance curves in Fig. 3*D* are too small or too fast to explain ice core variability on their own, while sources or sinks that lie near the required curves can account for a substantial fraction of the variance. For example, Zhang et al. (ref. 48) estimate that modern interannual variability in wetland emissions is 4.7 Tg CH_4_/y from an ensemble of process-based models (i.e., $\sigma_{\varepsilon }^{2}=22$ [Tg CH_4_/y]^2^; corresponding to $\tau_{\varepsilon }≳1$ y). Together with the OH variability discussed above (Fig. 2*I*), these fast-varying components are likely sufficient to reproduce the observed ice core methane variance in our framework, even before considering other sources or sinks such as biomass burning. Shaded patches in Fig. 3*D* show approximate parameter spaces ($\tau_{\varepsilon }$ vs. $\sigma_{\varepsilon }^{2}$) of OH oxidation, wetland methane emissions, and biomass burning methane emissions derived from inventories or simulations of modern methane variability (*SI Appendix*, section 3). Within these ranges, plausible combinations can reproduce the observed ice core variance, though structural uncertainty in variability estimates remains large.

No coupled process-based model of methane sources and sinks is currently calibrated for methane internal variability. A clear next step is to jointly constrain source–sink dynamics within a coupled model so that the covariance of the combined sources and sinks is consistent with observed ice core methane variability. Similar ice core methane variability to the WAIS Divide record has also been observed in deeper-time records (24, 25), which provide additional opportunity to evaluate source and sink parameterizations in different background climates. In practice, calibrating such a model may require information beyond bulk methane, including methane isotopes (e.g., refs. 15, 23, and 30) and other tracers (e.g., CO, CO_2_) that carry source- or sink-specific signatures.

## Conclusions

Simple models of preindustrial methane variability that treat the source–sink imbalance as a random process show that a null hypothesis of unforced dynamics can reproduce variability observed in the ice core record due to smoothing by the methane lifetime and firn processes. Our framework provides a baseline for hypothesis testing submillennial excursions in the ice core record: Small excursions that are reproduced by the null hypothesis do not require synchronous forcing to be explained, whereas larger and faster excursions that depart from this baseline variability such as the industrial transition or abrupt climate events (e.g., the Younger Dryas) require additional forcing. Given that these simple models reproduce ice core variability with few (two to three) parameters, attribution of small methane excursions to forcing requires strong process-based evidence. Such frameworks could be explored for records of other short-lived trace gases (e.g., ref. 49).

By extending the white noise null hypothesis to a physically interpretable parameter space of source–sink imbalance timescales and magnitudes, we show that a broad range of source–sink timescales can reproduce ice core methane variability. Fast-varying sources and sinks, previously considered too small or transient to affect ice core methane, may substantially contribute to variability in the observed record. These fast dynamics serve to amplify unforced atmospheric methane variability. A key consequence is that fast-varying sources or sinks could also explain modern methane growth rate variability. As such, constraining the dynamics of methane sources and sinks with preindustrial ice core records could provide constraints on modern methane trends. Observations of preindustrial methane from higher-accumulation sites would be useful to reduce firn smoothing and better characterize high-frequency variability (*SI Appendix*, Fig. S10).

## Materials and Methods

### Model for Submillennial Methane Variability.

The continuity equation for the atmospheric methane burden is

[3] $$\begin{array}{c} \frac{\mathrm{dC}}{\mathrm{dt}}=S-kC. \end{array}$$

The tendency of methane concentrations (dC/dt) is governed by a balance of its sources (S) and sinks (kC), in which k is a first-order rate coefficient for methane loss ($k=\tau^{-1}$) encapsulating atmospheric oxidation and other minor methane sinks (e.g., uptake from soils). Perturbations to the methane burden are proportional to perturbations in globally averaged mixing ratio. In the main text, we express methane loss in time units (τ) for ease of interpretation as an e-folding time. Here, we use k for convenience in the derivation of the linear model (Eq. **1**). Using τ instead of k is equivalent and trivial. We define perturbations to methane concentrations, sources, and loss rate coefficient as $C=\bar{C}+C^{\prime }$, $S=\bar{S}+S^{\prime }$, and $k=\bar{k}+k^{\prime }$. Methane sinks (kC) can be linearized to first order:

[4] $$\begin{array}{c} kC\approx \bar{k}\;\bar{C}+\bar{k}\;C^{\prime }+k^{\prime }\;\bar{C}. \end{array}$$

Perturbations to the loss rate ($k^{\prime }$) can be decomposed into the coupled chemical response (feedback) to a methane perturbation (dk/dC) (32, 33) and perturbations exogenous to the atmospheric methane system from internal climate variability or external forcings ($k_{\text{exo.}}^{\prime }$):

[5] $$\begin{array}{c} k^{\prime }={\left.\frac{\mathrm{dk}}{\mathrm{dC}}\right|}_{\bar{C}}C^{\prime }+k_{\text{exo.}}^{\prime }. \end{array}$$

Noting that $\bar{S}=\bar{k}\;\bar{C}$, substituting Eq. **5** into Eq. **4** and the result into Eq. **3** gives

[6] $$\begin{array}{c} \frac{dC^{\prime }}{\mathrm{dt}}=S^{\prime }-\left(\bar{k}+{\left.\frac{\mathrm{dk}}{\mathrm{dC}}\right|}_{\bar{C}}\;\bar{C}\right)C^{\prime }-k_{\text{exo.}}^{\prime }\;\bar{C}. \end{array}$$

The methane source–sink imbalance is defined as the net perturbation to the sources and sinks of methane:

[7] $$\begin{array}{c} \varepsilon \equiv S^{\prime }-k_{\text{exo.}}^{\prime }\;\bar{C}. \end{array}$$

Because $k^{\prime }={\left.\frac{\mathrm{dk}}{d\tau }\right|}_{\bar{\tau }}\tau^{\prime }$, exogenous perturbations to the rate coefficient can be expressed as $k_{\text{exo.}}^{\prime }=-\tau_{\text{exo.}}^{\prime }/{\bar{\tau }}^{2}$. If reexpressing methane sinks in terms of a lifetime perturbation, Eq. **7** becomes $\varepsilon \equiv S^{\prime }+\tau_{\text{exo.}}^{\prime }\bar{C}/{\bar{\tau }}^{2}\;$.

Following Holmes (33), we express the linearized first-order methane loss as a steady-state rate constant ($\bar{k}$) and feedback coefficient:

[8] $$\begin{array}{c} R\equiv {\left.\frac{\mathrm{dk}}{\mathrm{dC}}\right|}_{\bar{C}}\frac{\bar{C}}{\bar{k}}. \end{array}$$

The perturbation lifetime of methane is therefore

[9] $$\begin{array}{c} \tau_{C^{\prime }}=\frac{1}{\left(1+R\right)\;\bar{k}}. \end{array}$$

An emission (perturbation) of methane titrates available oxidants, decreasing the loss rate of methane (R<0) and increasing its perturbation lifetime relative to its steady-state lifetime ($\tau_{C^{\prime }}>\bar{\tau }$). Substituting the definitions of ε (Eq. **7**) and $\tau_{C^{\prime }}$ (Eq. **9**) into Eq. **6** gives the perturbation model for atmospheric methane (Eq. **1**).

### Firn Air Model.

We use a firn air smoothing kernel (gas age distribution) for the WAIS Divide WDC06A core (*SI Appendix*, Fig. S9) produced by the Center for Ice and Climate, University of Copenhagen, firn air transport model (40), which simulates molecular diffusion, deep firn eddy mixing, and bubble trapping and compaction (50) with an advection–diffusion equation. The age distribution is spectrally white at frequencies higher than 1/10 y^−1^, though our conclusions are not sensitive to this spectral feature. We note that the WDC06A gas age distribution has been published previously (50) and was used unaltered for this work.

### Solutions for Expected Variance of Atmospheric Methane and Source–Sink Imbalance.

Solutions for the relationship between ice core variance, expected atmospheric methane variance, and expected source–sink imbalance variance can be derived from Eqs. **1** and **2**. Given that the system is linear and forced by Gaussian white noise, Eqs. **1** and **2** can be expressed as a multivariate Ornstein–Uhlenbeck process:

[10] $$\begin{array}{cc} dx & =Axdt+BdW,\\ x=\left[\begin{array}{c} C^{\prime }\\ \varepsilon \end{array}\right],\;A & =\left[\begin{array}{cc} -\tau_{C^{\prime }}^{-1} & 1\\ 0 & -\tau_{\varepsilon }^{-1} \end{array}\right],\;B=\left[\begin{array}{c} 0\\ \sigma_{\eta } \end{array}\right]. \end{array}$$

In this form, the Gaussian white noise process η in Eq. **2** is implemented with the Wiener process dW, which represents the integral of a Gaussian white noise process scaled by the innovation SD ($\sigma_{\eta }$).

Since the matrix A is stable (eigenvalues have negative real parts), the steady-state instantaneous covariance of the system (Σ) can be solved with the fluctuation–dissipation relationship (51, 52):

[11] $$\begin{array}{c} A\Sigma +\Sigma A^{T}+BB^{T}=0,\;\Sigma =\left[\begin{array}{cc} \sigma_{C^{\prime }}^{2} & \sigma_{C^{\prime },\varepsilon }\\ \sigma_{C^{\prime },\varepsilon } & \sigma_{\varepsilon }^{2} \end{array}\right]. \end{array}$$

Solving the elements of Eq. **11** gives the variance of atmospheric methane ($\sigma_{C^{\prime }}^{2}$) as a function of $\tau_{C^{\prime }}$, $\tau_{\varepsilon }$, and $\sigma_{\varepsilon }^{2}$:

[12] $$\begin{array}{c} \sigma_{C^{\prime }}^{2}=\frac{\tau_{C^{\prime }}^{2}\tau_{\varepsilon }}{\tau_{C^{\prime }}+\tau_{\varepsilon }}\;\sigma_{\varepsilon }^{2}. \end{array}$$

The expected “variance inflation ratio” between atmospheric methane anomalies ($\sigma_{C^{\prime }}^{2}$) and ice core methane anomalies ($\sigma_{\text{ice core}}^{2}$) can be derived from the firn smoothing kernel and the autocorrelation function of atmospheric methane anomalies (see *SI Appendix*, section 1 for the full derivation):

[13] $$\begin{array}{c} \frac{\sigma_{C^{\prime }}^{2}}{\sigma_{\text{ice core}}^{2}}={\left(q\left(0\right)+2\sum_{n\geq 1}q\left(n\right)\rho_{C^{\prime }}\left(n\right)\right)}^{-1}. \end{array}$$

Let h(n) be the normalized discrete firn smoothing kernel at steps n of time interval Δt (here, 0.5 y) such that $\sum_{n}h\left(n\right)=1$. The self-overlap function of the firn kernel is therefore $q\left(n\right)=\sum_{k\geq 0}h\left(k\right)h\left(k+n\right)$. $\rho_{C^{\prime }}\left(n\right)$ is the autocorrelation function of atmospheric methane anomalies at lags of n. Because the firn kernel is normalized, the expression in ${\left(\cdot \right)}^{-1}$ in Eq. **13** approaches one as timescales increase, which cause the autocorrelation of atmospheric methane to increase (i.e., atmospheric variability is low-frequency).

## Supplementary Material

Appendix 01 (PDF)

## Data Availability

Code and data have been deposited in a repository at https://doi.org/10.5281/zenodo.18463579 (53).

## Footnotes

^*^We note that we have increased the OH variability by a factor of 6.5. Murray et al. (ref. 35) note that OH variability in this generation of CMIP5 models (including GFDL-CM3) is biased low relative to modern estimates of OH variability (44).
