# Supplementary material for: Multidecadal preindustrial methane variability can be explained by noise in the source–sink imbalance
Source: Proc Natl Acad Sci U S A. 2026 Jun 15;123(25):e2601235123. doi: 10.1073/pnas.2601235123 (PMC13291627; doi:10.1073/pnas.2601235123)
Supplement: Supplementary file 1 — Appendix 01 (PDF) [file pnas.2601235123.sapp.pdf]

Supporting Information for

Multidecadal preindustrial methane variability can be explained by noise in the source–sink imbalance

Eric J. Mei, Gregory J. Hakim, Cristian Proistosescu, Thomas K. Bauska, Christo Buizert, and Alexander J. Turner

Eric J. Mei
E-mail: emei@uw.edu

This PDF file includes:

Supporting text
Figs. S1 to S11
Table S1
SI References

Supporting Information Text

S1. Derivation of expected ratio of atmospheric to ice core methane variance

Here, we derive the solutions for the expected ratio of the atmospheric methane variance to the ice core methane variance ("variance inflation ratio" in the main text). Perturbation equations in the main text for atmospheric methane and the budget imbalance are reproduced below:

$$\frac{dC'}{dt} = -\tau_{C'}^{-1} C' + \varepsilon, \qquad [1]$$

$$\frac{d\varepsilon}{dt} = -\tau_{\varepsilon}^{-1} \varepsilon + \eta. \qquad [2]$$

Though we define Eq. 1 and 2 in continuous time, we approach the derivation of the variance inflation ratio with a discrete time approach to be consistent with the discrete firn smoothing filter. In hindsight, a more elegant derivation would be to use a continuous time approach to take advantage of the Fourier transform and avoid the time-domain convolutions in the approach here. Let $\Delta t = 0.5$ years be the uniform time step that defines the index n for the empirical firn smoothing kernel $h(n)$, which is normalized such that $\sum_n h(n) = 1$. $y(t)$ is the firn-smoothed atmospheric methane anomaly $C'(t)$ obtained by convolving the atmospheric signal with the firn smoothing kernel:

$$y(t) = \sum_{n \geq 0} h(n)\, C'(t - n\Delta t) \qquad [3]$$

Note again that $y(t)$ and $C'(t)$ are defined such that t is restricted to observations of the underlying continuous system at multiples of Δt.

The autocovariance of atmospheric methane $\gamma_{C'}(n)$ can be defined as

$$\gamma_{C'}(n) \equiv \mathbb{E}\left[C'(t)\, C'(t + n\Delta t)\right], \quad \sigma_{C'}^2 = \gamma_{C'}(0), \qquad [4]$$

in which $\mathbb{E}\left[\cdot\right]$ indicates an expectation. The covariance of y, $\sigma^2_{\text{ice core}}$, can be derived from

$$\begin{aligned} \sigma^2_{\text{ice core}} &= \mathbb{E}\left[y(t)^2\right] \\ &= \mathbb{E}\left[\left(\sum_{k \geq 0} h(k) C'(t - k\Delta t)\right)\left(\sum_{\ell \geq 0} h(\ell) C'(t - \ell\Delta t)\right)\right] \\ &= \sum_{k \geq 0}\sum_{\ell \geq 0} h(k) h(\ell)\, \mathbb{E}\left[C'(t - k\Delta t)\, C'(t - \ell\Delta t)\right]. \end{aligned} \qquad [5]$$

Define the self-overlap of the firn kernel as

$$q(n) \equiv \sum_{k \geq 0} h(k)\, h(k + n). \qquad [6]$$

By performing a change of variables $n = \ell - k$, time indices of C' in the expectation $\mathbb{E}\left[\cdot\right]$ of Eq. 5 can be re-expressed as $(t - k\Delta t) - (t - \ell\Delta t) = (\ell - k)\Delta t$. Thus, $\mathbb{E}\left[C'(t - k\Delta t)\, C'(t - \ell\Delta t)\right] = \gamma(n)$ because the system is stationary. Substituting this expression and Eq. 6 into Eq. 5 gives

$$\begin{aligned} \sigma^2_{\text{ice core}} &= \sum_{n=-\infty}^{\infty} q(n)\, \gamma_{C'}(n) \\ &= \sigma_{C'}^2 \left(q(0) + 2\sum_{n \geq 1} q(n)\, \rho_{C'}(n)\right). \end{aligned} \qquad [7]$$

$\rho_{C'}(n)$ is the autocorrelation of C' at lags of n, defined as $\rho_{C'}(n) = \gamma_{C'}(n)/\sigma^2_{C'}$. Note that Eq. 7 is a rearrangement of Eq. 13 in the main text.

Autocorrelation of atmospheric methane. The two-timescale methane perturbation model (Eq. 1 and 2) can be written as a cascade of two discrete AR(1) processes driven by white noise η_t:

$$C'_t = \phi_{C'}\, C'_{t-1} + \varepsilon_t, \qquad [8]$$

$$\varepsilon_t = \phi_{\varepsilon}\, \varepsilon_{t-1} + \eta_t. \qquad [9]$$

Let $g_\phi[k]$ denote the response of a single AR(1) process at lag k to a unit perturbation:

$$g_\phi[k] = \phi^k, \qquad [10]$$

so that the overall impulse response from η to C' is the convolution

$$g = g_{\phi_{C'}} * g_{\phi_\varepsilon}, \qquad C'_t = \sum_{k\geq 0} g[k]\,\eta_{t-k}. \tag{11}$$

Expanding the convolution gives

$$g[k] = \sum_{j=0}^{k} g_{\phi_{C'}}[k-j]\, g_{\phi_\varepsilon}[j] = \sum_{j=0}^{k} \phi_{C'}^{k-j}\phi_\varepsilon^{j} = \phi_{C'}^{k} \sum_{j=0}^{k} \left(\frac{\phi_\varepsilon}{\phi_{C'}}\right)^{j}. \tag{12}$$

Using the finite geometric-series identity $\sum_{j=0}^{k} r^j = \frac{1-r^{k+1}}{1-r}$ for $|r|<1$,

$$g[k] = \frac{\phi_{C'}^{k+1} - \phi_\varepsilon^{k+1}}{\phi_{C'} - \phi_\varepsilon}, \qquad k \geq 0, \;\; \phi_{C'} \neq \phi_\varepsilon. \tag{13}$$

From Eq. 13,

$$\gamma_{C'}(\tau) = \mathbb{E}[C'_t\, C'_{t+\tau}] = \sigma_\eta^2 \sum_{n\geq 0} g[n]\, g[n+|\tau|], \tag{14}$$

since η_t is white noise with $\mathbb{E}[\eta_t \eta_{t'}] = \sigma_\eta^2$ if $t = t'$.

Substituting Eq. 13 into Eq. 14 and expanding,

$$\gamma_{C'}(\tau) = \frac{\sigma_\eta^2}{(\phi_{C'} - \phi_\varepsilon)^2} \sum_{n\geq 0} \Big[\phi_{C'}^{2n+|\tau|+2} - \phi_\varepsilon^{n+1}\phi_{C'}^{n+|\tau|+1} - \phi_{C'}^{n+1}\phi_\varepsilon^{n+|\tau|+1} + \phi_\varepsilon^{2n+|\tau|+2}\Big]. \tag{15}$$

Using $\sum_{n\geq 0} r^n = (1-r)^{-1}$ for $|r|<1$,

$$\sum_{n\geq 0} \phi_{C'}^{2n+|\tau|+2} = \phi_{C'}^{|\tau|+2}(1-\phi_{C'}^2)^{-1}, \tag{16}$$

$$\sum_{n\geq 0} \phi_\varepsilon^{n+1}\phi_{C'}^{n+|\tau|+1} = \phi_\varepsilon \phi_{C'}^{|\tau|+1}(1-\phi_\varepsilon\phi_{C'})^{-1}, \tag{17}$$

$$\sum_{n\geq 0} \phi_{C'}^{n+1}\phi_\varepsilon^{n+|\tau|+1} = \phi_{C'} \phi_\varepsilon^{|\tau|+1}(1-\phi_{C'}\phi_\varepsilon)^{-1}, \tag{18}$$

$$\sum_{n\geq 0} \phi_\varepsilon^{2n+|\tau|+2} = \phi_\varepsilon^{|\tau|+2}(1-\phi_\varepsilon^2)^{-1}. \tag{19}$$

Substituting these into Eq. 15 yields

$$\boxed{\gamma_{C'}(\tau) = \frac{\sigma_\eta^2}{(\phi_{C'} - \phi_\varepsilon)^2}\left[\frac{\phi_{C'}^{|\tau|+2}}{1-\phi_{C'}^2} - \frac{\phi_\varepsilon\phi_{C'}^{|\tau|+1} + \phi_{C'}\phi_\varepsilon^{|\tau|+1}}{1-\phi_{C'}\phi_\varepsilon} + \frac{\phi_\varepsilon^{|\tau|+2}}{1-\phi_\varepsilon^2}\right], \quad |\phi_{C'}|, |\phi_\varepsilon| < 1.} \tag{20}$$

Let $S(\tau)$ denote the bracketed term ($[\cdot]$). The autocorrelation is then

$$\boxed{\rho_{C'}(\tau) = \frac{\gamma_{C'}(\tau)}{\gamma_{C'}(0)} = \frac{S(\tau)}{S(0)}.} \tag{21}$$

Equal poles solution. If $\phi_{C'} = \phi_\varepsilon = \phi$, the impulse response of two AR(1) processes (Eq. 11) with the same 1-lag autocorrelation ϕ is

$$\begin{aligned} g[k] &= \sum_{j=0}^{k} \phi^{k-j}\phi^{j} \\ &= (k+1)\phi^{k}, \qquad k \geq 0. \end{aligned} \tag{22}$$

Substituting into Eq. 14 gives

$$\gamma_{C'}(\tau) = \sigma_\eta^2 \sum_{n\geq 0} (n+1)(n+|\tau|+1)\,\phi^{2n+|\tau|}. \tag{23}$$

Set $r \equiv \phi^2$. Expanding the terms of Eq. 23:

$$\gamma_{C'}(\tau) = \sigma_\eta^2 \phi^{|\tau|} \sum_{n\geq 0} (n+1)(n+|\tau|+1)\, r^2 \qquad [24]$$

$$= \sigma_\eta^2 \phi^{|\tau|} \left(\sum_{n\geq 0} n^2 r^n + (|\tau|+2) \sum_{n\geq 0} n r^n + (|\tau|+1) \sum_{n\geq 0} r^n \right). \qquad [25]$$

Because $r < 1$, the infinite geometric series identities $\sum_{n\geq 0} r^n = (1-r)^{-1}$, $\sum_{n\geq 0} n r^n = r(1-r)^{-2}$, and $\sum_{n\geq 0} n^2 r^n = r(1+r)(1-r)^{-3}$ can be used:

$$\gamma_{C'}(\tau) = \sigma_\eta^2\, \phi^{|\tau|}\, \frac{(|\tau|+1) + (1-|\tau|)\phi^2}{(1-\phi^2)^3}, \qquad |\phi| < 1. \qquad [26]$$

The variance and autocorrelation are therefore

$$\gamma_{C'}(0) = \sigma_\eta^2\, \frac{1+\phi^2}{(1-\phi^2)^3}, \qquad [27]$$

$$\boxed{\rho_{C'}(\tau) = \phi^{|\tau|}\, \frac{(|\tau|+1) + (1-|\tau|)\phi^2}{1+\phi^2}.} \qquad [28]$$

S2. Irregular sampling, spectral artifacts, and the Nyquist frequency

The spectral window ($W(f)$) created by the irregular sampling of the data can be calculated with a Fourier transform of a Dirac delta comb with Dirac delta functions at the spacing (t_i) of the observations:

$$W(f) = \frac{1}{N} \left| \sum_{i=1}^{N} e^{-2\pi i f t_i} \right|^2 \qquad [29]$$

Fig. S4 shows the spectral window of all cores investigated (WDC05A, WDC06A, GISP2, and NEEM). Fig. S2 shows a version of main text Fig. 1 for all cores. Because sampling is a multiplication of a Dirac delta comb in the time domain, the true spectrum of the underlying continuous process is convolved with $W(f)$ in the frequency domain. Fig S5 shows the effect of the sampling window on redder and redder continuous processes: as the noise becomes redder, the power spectra of the sampled data converge to the sampling window.

Note that the standard Nyquist sampling limit ("Nyquist frequency"; $f_{\mathrm{Ny}} = 1/2\Delta t$) for periodograms of uniformly sampled signals does not have a neat companion for non-uniformly sampled signals. Periodograms derived from non-uniformly sampled signals can correctly identify spectral features far beyond the standard Nyquist frequency (e.g., Fig. 11 from ref. 1). The Nyquist frequency for non-uniformly sampled signals does not exist if the ratio between any pair of sampling intervals is irrational (2), and therefore the highest frequency shown for a Lomb-Scargle periodogram is somewhat arbitrary in practice. We use an implementation of the Lomb-Scargle periodogram from the Astropy package (3), which by default sets an upper frequency limit as 2.5 times the frequency of the mean sampling interval (for WDC06A, $f_{\mathrm{upper}} \approx 0.2$ years^{-1}). We retain this upper frequency limit in Fig. 1 shown in the main text because 1) artifacts from the spectral window exist at all frequencies (Fig. S5), even at frequencies lower than any supposed Nyquist frequency; and 2) our forward modeling (firn smoothing, non-uniform sampling) applies the same artifacts to our simulations, which mitigates unbalanced interpretation of observations compared to our simulations.

S3. Estimates of source–sink imbalance parameter space

Very few general circulation models simulate a full methane cycle, so methane's sources and sinks have large structural uncertainty. To arrive at a first order approximation of the plausible parameter space in Fig. 3d in the main text, we rely on estimates of methane budget variability from a mixture of inventories, process-based model simulations, and top-down inversions. Table S1 shows the variance of methane budget perturbations from OH oxidation (4–6), biomass burning emissions (7, 8), and wetland emissions (9, 10). Note that these estimates are of *modern* methane variability. Biomass burning emissions considered here are only from methane emission itself, and do not include reactive nitrogen or non-methane carbon emissions that could result in higher-order feedbacks on the source–sink imbalance due to changes to methane oxidants (11). While not considered here, these feedbacks in theory could be represented in the OH reconstructions presented. Variability in wetland emissions are largely uncertain, with variance from the WetCHARTS ensemble (10) spanning an order of magnitude due to differences in low-frequency variability between ensemble members. Similarly, models in the WetCHIMP intercomparison (9) also span an order of magnitude in variance.

Note that estimates from Turner et al. (ref. 5) and Rigby (ref. 6) are not process-based, and therefore have few constraints on dynamics of OH. Estimates of OH variability from methyl chloroform presented by Montzka et al. (ref. 4) were originally presented in percentage anomalies of OH relative to 2000-2005. To translate percentage OH anomalies into Tg CH_4/year, we

used the scaling presented by Turner et al. (ref. 8), who tuned the anomalies by a factor needed to explain the methane hiatus. These three estimates of variability are also non-unique, since Turner et al. (ref. 5) and Rigby (ref. 6) use methyl chloroform measurements presented by Montzka et al. (ref. 4) to infer OH variability.

Estimates of timescale are approximate (Fig. S11) and difficult to directly quantify due to 1) short simulation or record length (often one to two decades) and 2) resolution of the simulations or records. Datasets averaged to monthly or annual resolution make it difficult to estimate the true dynamic timescale of variability, especially if the timescale is shorter than the averaging resolution. Future efforts could interpret these datasets at finer resolution or use statistical methods (e.g., Gaussian process models) to recover the timescale of source or sink variability. Such efforts are necessary given the power law scaling in Fig. 3d in the main text at timescales below 10 years. It is also possible that other statistical models of noise different than the ones we present (in Eq. 2 in the main text) better approximate the true dynamics of sources and sinks. These models may not fit neatly in the framework we propose in Fig. 3d in the main text.

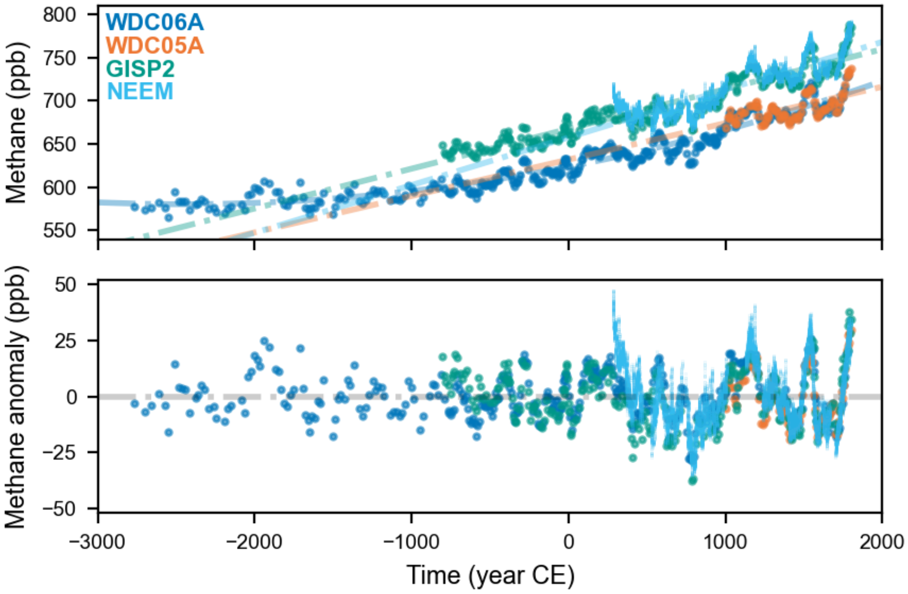

Fig. S1. Timeseries of all ice core records used for analysis: WDC06A, WDC05A, GISP2 (12), and NEEM (13). (*top*) Methane concentrations (points) from each core along with the fitted trend (dashed line). First-order polynomials were used in all cores except for WDC06A, which used a second-order polynomial. (*bottom*) Detrended methane anomalies from each core. Dashed line indicates anomaly of zero ppb.

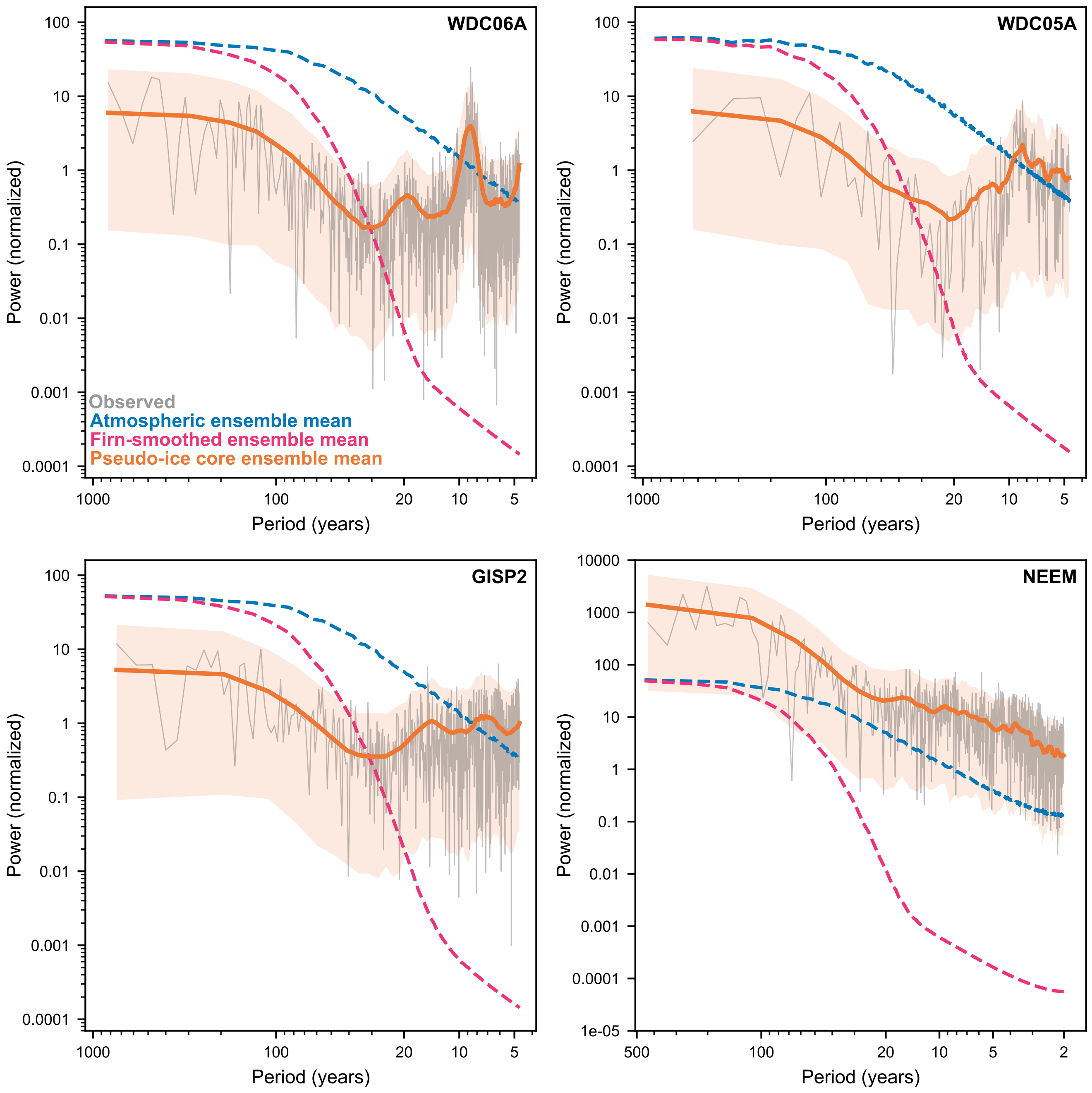

Fig. S2. Same as Fig. 1 in the main text but for all ice core records analyzed. Figure for WDC06A is reproduced from Fig. 1b in the main text. We use the WDC06A record for its high frequency observations over the last millennium and coverage extending to nearly 5,000 years before present (n=389, mean sampling frequency of once per 11.7 years), which gives a more robust estimate of sub-millennial spectral features compared to other records. Note that the NEEM record has a long high-frequency portion that is truncated.

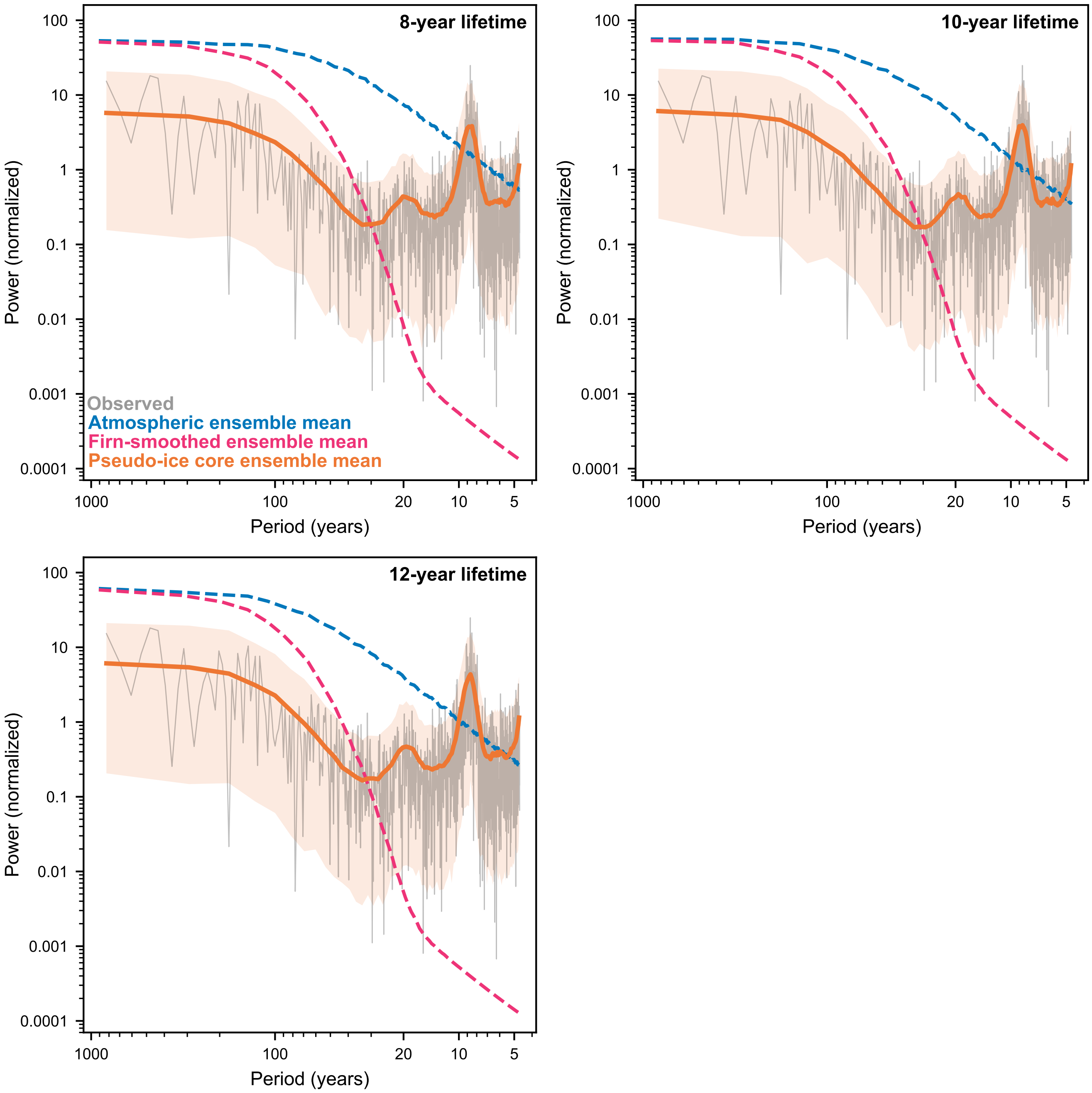

Fig. S3. Same as Fig. 1 in the main text but for methane perturbation lifetimes of 8, 10, and 12 years. 10-year lifetime used in the main text.

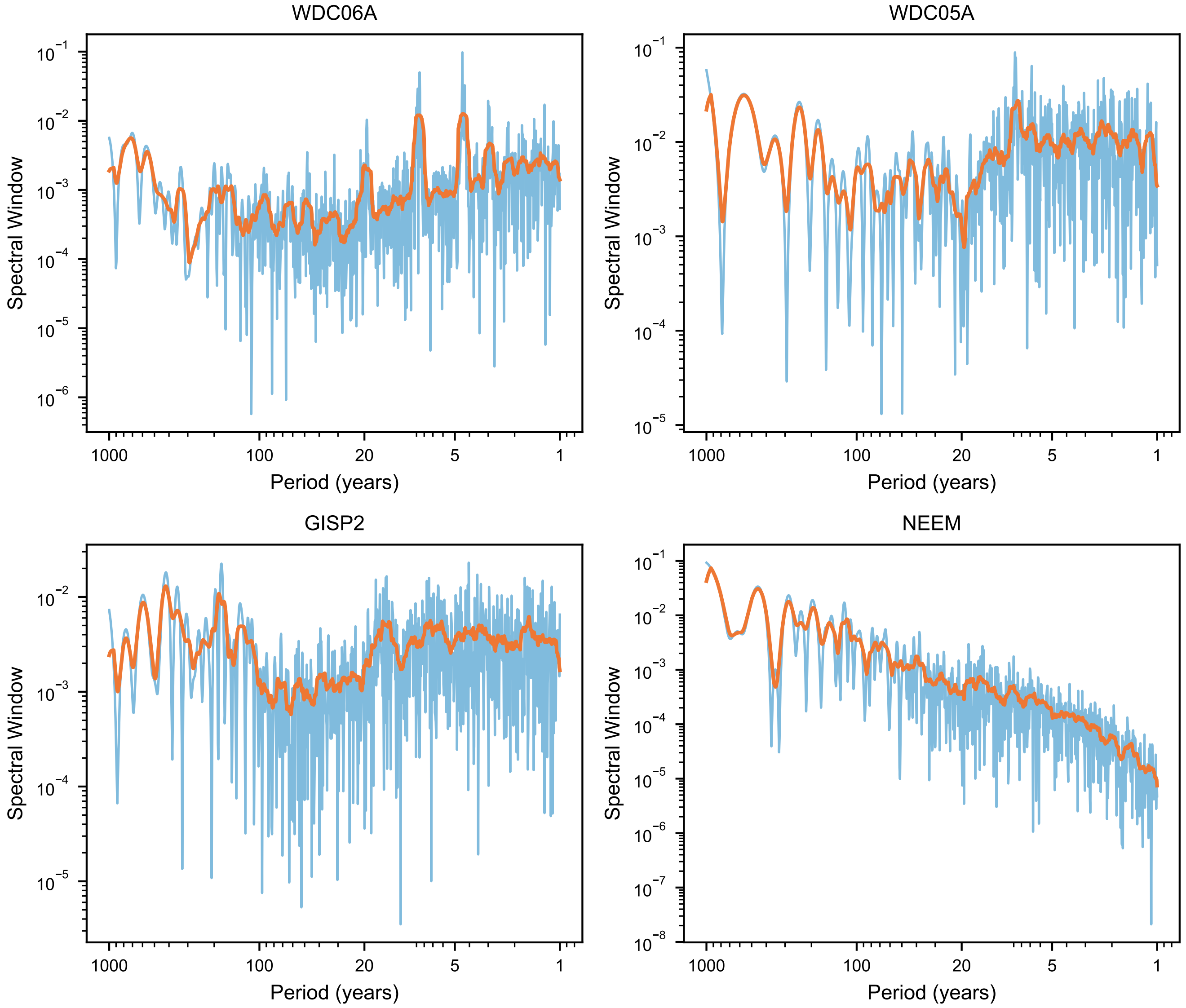

Fig. S4. Spectral windows in the frequency domain caused by irregular sampling for all ice core records analyzed. Orange line is a 20-frequency running mean of the blue line.

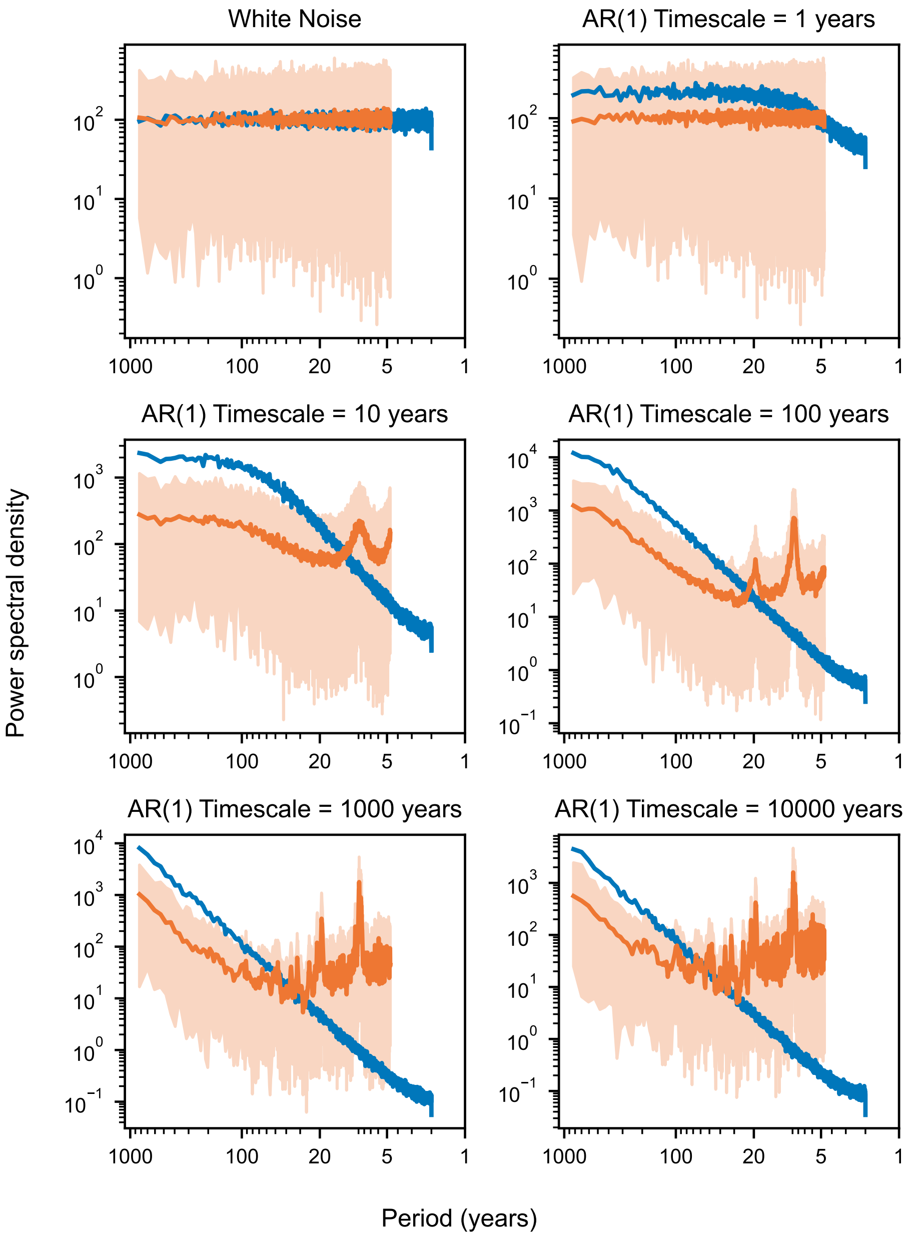

Fig. S5. Spectral leakage artifacts imposed by the spectral window of the WDC06A ice core (Fig. S4) as the decorrelation timescale of red noise increases. Blue line is the 1,000-member ensemble mean of the power spectrum of a white noise or AR(1) process sampled uniformly at 1-year intervals. Orange line is the 1,000-member ensemble mean of the power spectrum of the same processes sampled at the same intervals as the WDC06A core. Shading indicates the 95% confidence interval. The evenly-sampled power spectrum is convolved with the spectral window. Note that as the timescale of the AR(1) increases, this convolution results in a convergence to the spectral window, limiting the utility of the power spectrum for constraining long decorrelation timescales.

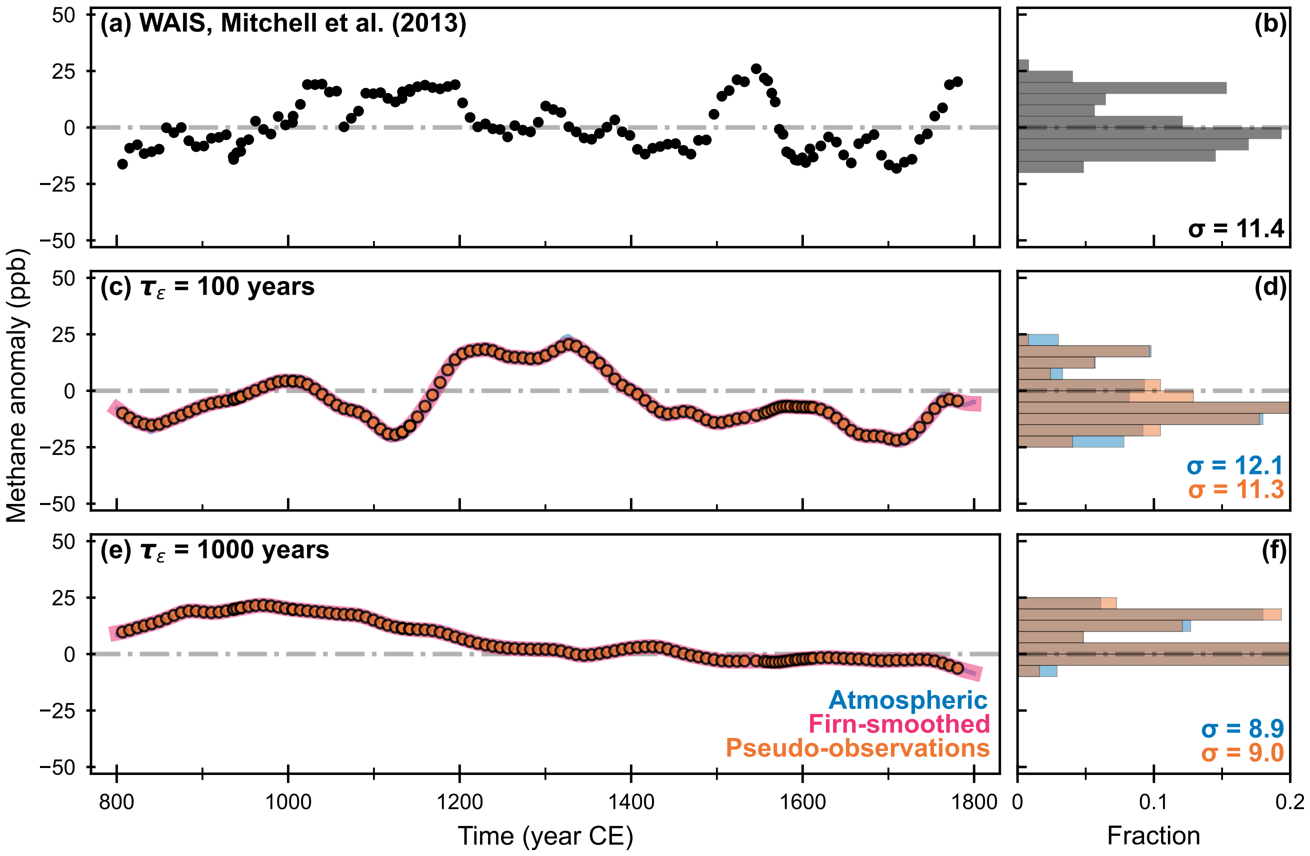

Fig. S6. Same as Fig. 2 in the main text but for source-sink imbalance timescales of 100 and 1,000 years. While the 100-year timescale simulation could plausibly be similar to observations with a little added noise, the 1,000-year timescale simulation varies too slowly to be similar to observations.

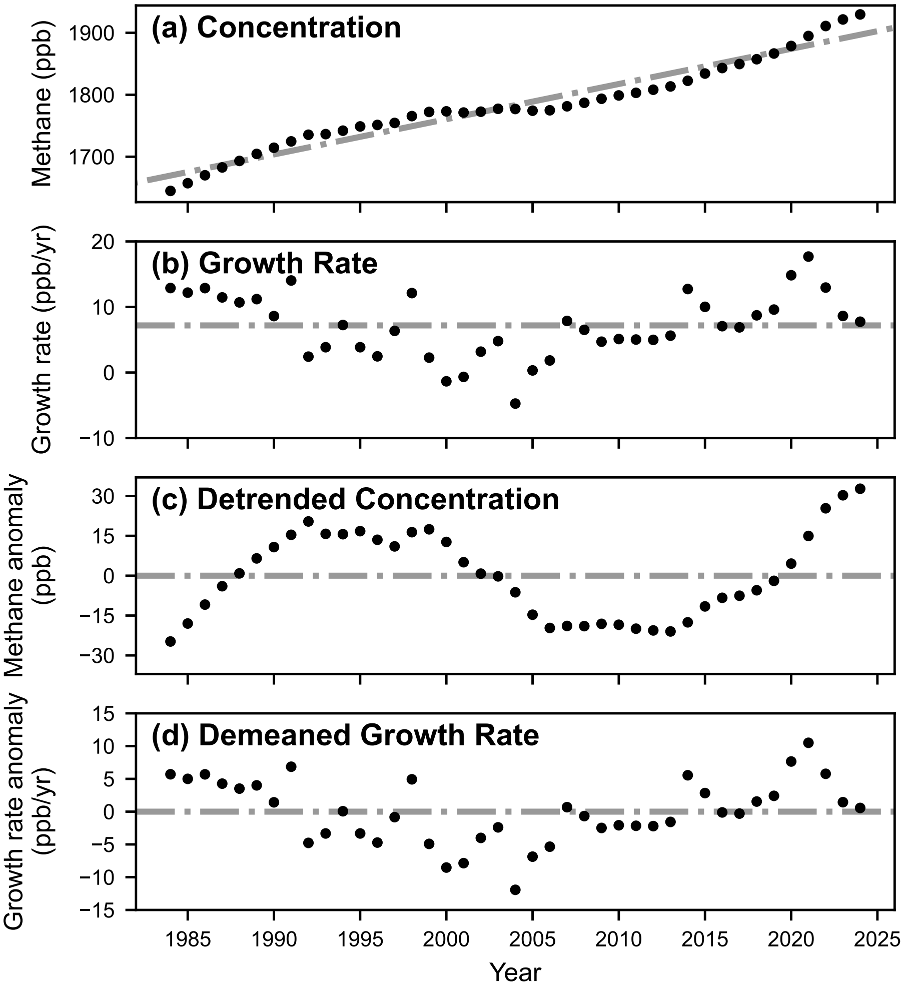

Fig. S7. Detrending of modern methane for the growth rate distribution shown in Fig. 3c in the main text. (*a*) Methane concentrations observed by NOAA flask network from 1984 to 2024 (14). Dashed line shows linear trend, which we take as the first-order estimate of the anthropogenic forced signal given the uncertainty in the attribution of higher-frequency variability (5, 6, 8). (*b*) Annual methane growth rates. Dashed line shows linear anthropogenic signal. (*c*) Methane concentrations detrended with linear trend. (*d*) Demeaned (detrended) annual methane growth rates, which are used to fit the distribution shown in Fig. 3c in the main text.

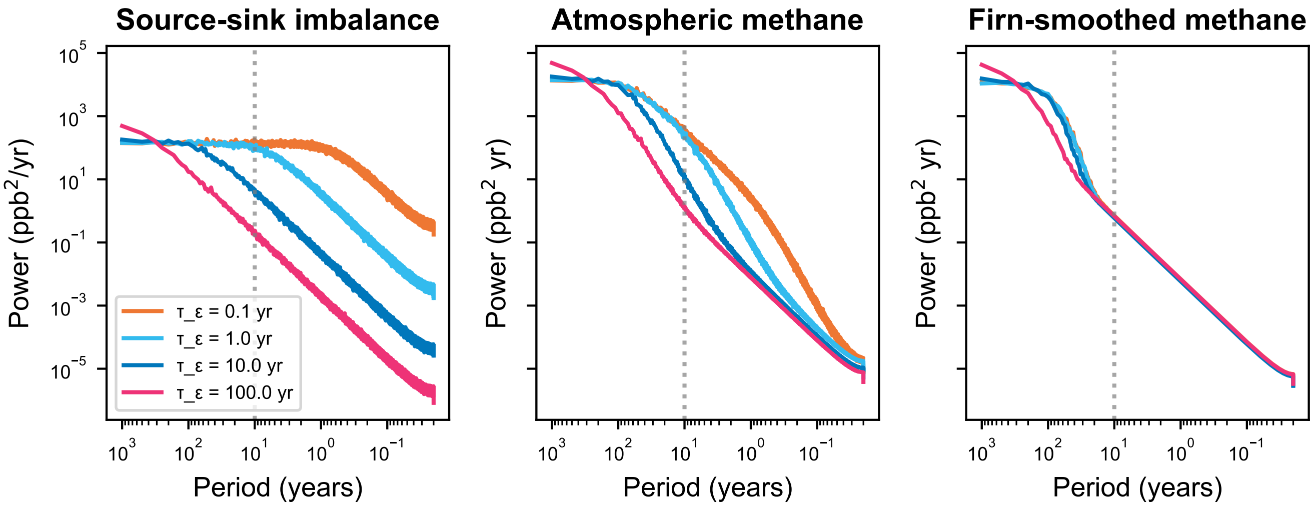

Fig. S8. Demonstration of increase in variance at short source–sink imbalance timescales. 100-member ensemble mean power spectra of source–sink imbalance (*left*), atmospheric methane (*middle*), and firn-smoothed methane (*right*) for order-of-magnitude increases in source–sink imbalance timescale from 0.1 years to 100 years. Vertical dotted line indicates methane lifetime (10 years). All signals are normalized such that the firn-smoothed signal has a variance of 100 ppb^2. As the timescale decreases, spectra of the firn-smoothed methane look more and more similar, but spectra of the source-sink imbalance differ. At short enough timescales (around 1 year), the low-frequency power in the source-sink imbalance converge, but the point at which power begins to taper moves to higher and higher frequencies. Thus, because the low-frequency power is preserved after smoothing by the methane lifetime and firn processes, pseudo-ice core methane records from source–sink imbalance timescales shorter than 0.1 years look statistically identical. However, per Parseval's theorem, the source-sink imbalance variance (area under the curves on the *left* figure) continues to increase as the timescale decreases.

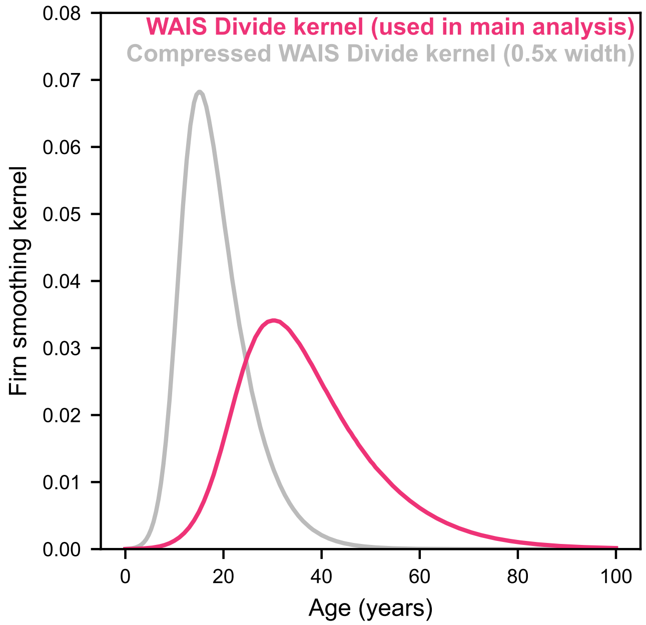

Fig. S9. Gas age distribution (firn smoothing kernel) for WAIS Divide WDC06A methane presented by Mitchell et al. (12). The original age distribution (magenta) was used in all analysis in the main text. A compressed age distribution (grey), defined as the original age distribution scaled by 0.5 in the time domain, is used to represent a hypothetical methane gas age distribution for a site with higher accumulation and therefore less firn smoothing for analysis in Fig. S10. Both age distributions have been normalized to unit area.

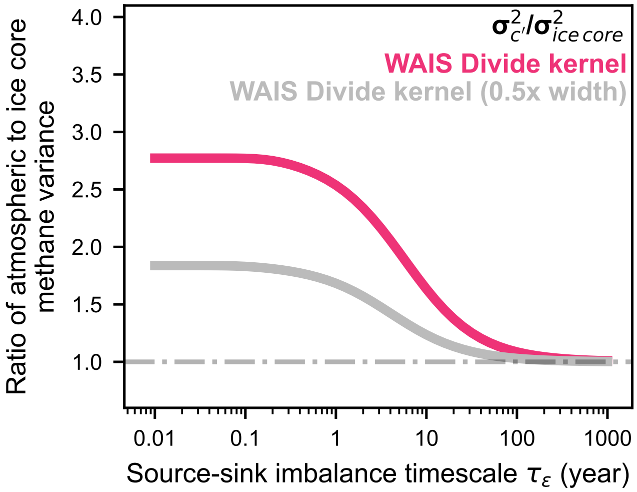

Fig. S10. Replica of Fig. 3a in the main text, which shows the expected ratio of atmospheric methane variance to ice core methane variance ("variance inflation ratio") as a function of the dominant source–sink imbalance timescale. The solution for $\tau_{C'} = 10$ years in the main text Fig. 3a is replicated (magenta). This solution ($\tau_{C'} = 10$) is re-derived with Eq. 13 in the main text for a compressed version of the WAIS Divide gas age distribution that is half the width of the original age distribution (grey; see Fig. S9). A core with less firn smoothing, perhaps because of higher accumulation at the core site, preserves more high-frequency atmospheric variability than the WDC06A core.

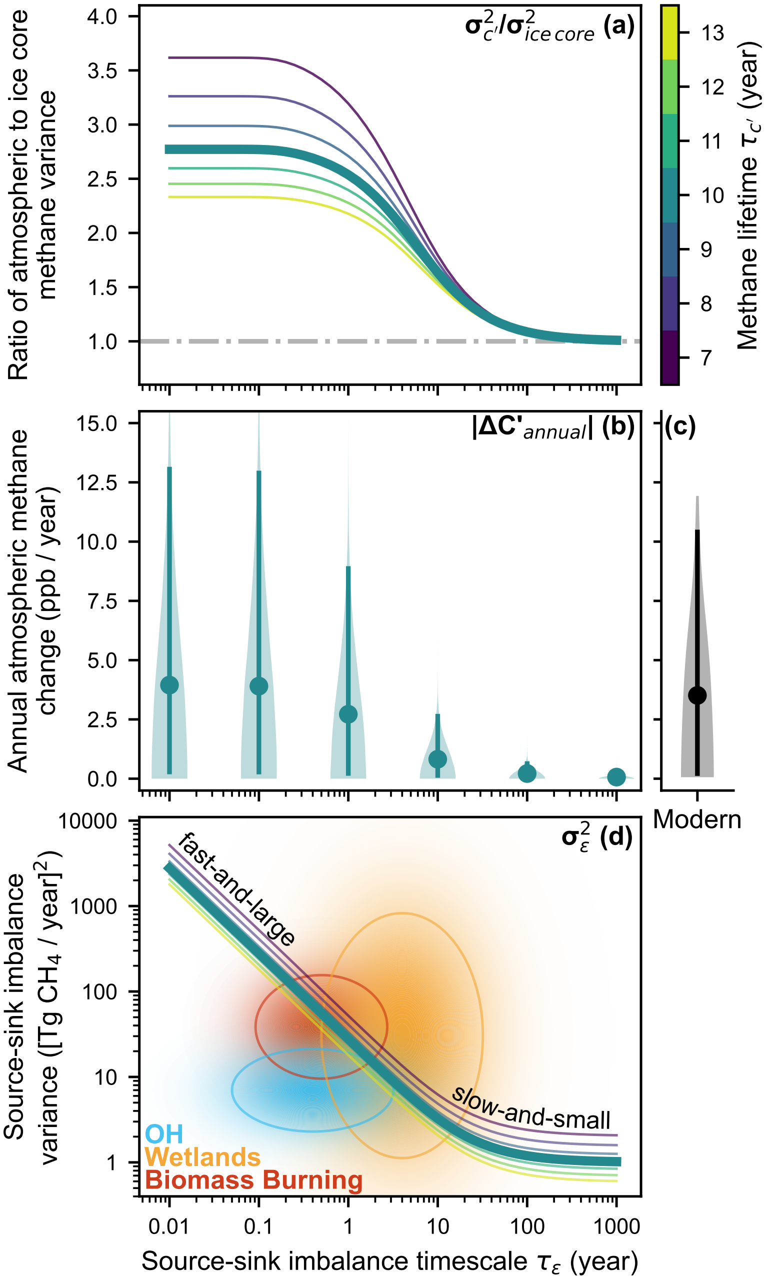

Fig. S11. Replica of Fig. 3 in the main text, with panel (*d*) updated to include solid lines alongside the shaded regions indicating the 2σ parameter space for OH oxidation (cyan), wetland methane emissions (orange), and biomass burning methane emissions (red).

Table S1. Variance of methane source–sink imbalance across datasets [Tg CH_4/year]^2

Dataset	Resolution	Variance	SE
Montzka et al. (ref. 4) OH	Annual	5.5	2.4
Turner et al. (ref. 5) OH	Annual	6.3	1.5
Rigby et al. (ref. 6) OH	Annual	9.9	2.4
GFED Biomass Burning (7)	Annual	31.0	10.1
EDGAR Biomass Burning (8)	Annual	47.9	11.1
WetCHARTs† ens. 1	Monthly	34.4	3.6
WetCHARTs ens. 2	Monthly	25.9	2.7
WetCHARTs ens. 3	Monthly	37.8	4.0
WetCHARTs ens. 4	Monthly	32.1	3.4
WetCHARTs ens. 5	Monthly	44.3	4.7
WetCHARTs ens. 6	Monthly	36.0	3.8
WetCHARTs ens. 7	Monthly	61.1	6.5
WetCHARTs ens. 8	Monthly	46.0	4.9
WetCHARTs ens. 9	Monthly	67.2	7.1
WetCHARTs ens. 10	Monthly	57.1	6.0
WetCHARTs ens. 11	Monthly	78.7	8.3
WetCHARTs ens. 12	Monthly	64.0	6.8
WetCHARTs ens. 13	Monthly	95.5	10.1
WetCHARTs ens. 14	Monthly	71.8	7.6
WetCHARTs ens. 15	Monthly	105.0	11.1
WetCHARTs ens. 16	Monthly	89.3	9.4
WetCHARTs ens. 17	Monthly	123.0	13.0
WetCHARTs ens. 18	Monthly	100.1	10.6
WetCHARTs ens. 1	Annual	5.9	2.2
WetCHARTs ens. 2	Annual	9.8	3.7
WetCHARTs ens. 3	Annual	12.6	4.8
WetCHARTs ens. 4	Annual	15.9	6.0
WetCHARTs ens. 5	Annual	17.4	6.6
WetCHARTs ens. 6	Annual	19.6	7.4
WetCHARTs ens. 7	Annual	10.5	4.0
WetCHARTs ens. 8	Annual	17.4	6.6
WetCHARTs ens. 9	Annual	22.4	8.5
WetCHARTs ens. 10	Annual	28.3	10.7
WetCHARTs ens. 11	Annual	30.9	11.7
WetCHARTs ens. 12	Annual	34.8	13.2
WetCHARTs ens. 13	Annual	16.4	6.2
WetCHARTs ens. 14	Annual	27.1	10.2
WetCHARTs ens. 15	Annual	34.9	13.2
WetCHARTs ens. 16	Annual	44.2	16.7
WetCHARTs ens. 17	Annual	48.3	18.3
WetCHARTs ens. 18	Annual	54.4	20.6
WetCHIMP‡ Bern	Monthly	760.1	89.9
WetCHIMP CLM4Me	Monthly	279.1	33.0
WetCHIMP DLEM	Monthly	195.9	23.2
WetCHIMP Orchidee	Monthly	563.3	66.6
WetCHIMP VIC	Monthly	47.0	5.6
WetCHIMP WSL	Monthly	65.7	7.8
WetCHIMP Bern	Annual	273.8	116.8
WetCHIMP CLM4Me	Annual	56.1	23.9
WetCHIMP DLEM	Annual	121.7	51.9
WetCHIMP Orchidee	Annual	227.6	97.1
WetCHIMP VIC	Annual	19.1	8.2
WetCHIMP WSL	Annual	25.3	10.8

† WetCHARTs ensemble of wetland methane emissions from Bloom et al. (ref. 10).
‡ WetCHIMP multi-model wetland methane inter-comparison from Melton et al. (ref. 9).

References

1. JT VanderPlas, Understanding the Lomb-Scargle Periodogram. *The Astrophys. J. Suppl. Ser.* **236**, 16 (2018).
2. L Eyer, P Bartholdi, Variable stars: Which Nyquist frequency? *Astron. Astrophys. Suppl. Ser.* **135**, 1–3 (1999).
3. The Astropy Collaboration, et al., The Astropy Project: Sustaining and Growing a Community-oriented Open-source Project and the Latest Major Release (v5.0) of the Core Package. *The Astrophys. J.* **935**, 167 (2022).
4. SA Montzka, et al., Small Interannual Variability of Global Atmospheric Hydroxyl. *Science* **331**, 67–69 (2011).
5. AJ Turner, C Frankenberg, PO Wennberg, DJ Jacob, Ambiguity in the causes for decadal trends in atmospheric methane and hydroxyl. *Proc. Natl. Acad. Sci.* **114**, 5367–5372 (2017).
6. M Rigby, et al., Role of atmospheric oxidation in recent methane growth. *Proc. Natl. Acad. Sci.* **114**, 5373–5377 (2017).
7. GR van der Werf, et al., Global fire emissions and the contribution of deforestation, savanna, forest, agricultural, and peat fires (1997–2009). *Atmospheric Chem. Phys.* **10**, 11707–11735 (2010).
8. AJ Turner, C Frankenberg, EA Kort, Interpreting contemporary trends in atmospheric methane. *Proc. Natl. Acad. Sci.* **116**, 2805–2813 (2019).
9. JR Melton, et al., Present state of global wetland extent and wetland methane modelling: Conclusions from a model inter-comparison project (WETCHIMP). *Biogeosciences* **10**, 753–788 (2013).
10. AA Bloom, et al., A global wetland methane emissions and uncertainty dataset for atmospheric chemical transport models (WetCHARTs version 1.0). *Geosci. Model. Dev.* **10**, 2141–2156 (2017).
11. LT Murray, AM Fiore, DT Shindell, V Naik, LW Horowitz, Large uncertainties in global hydroxyl projections tied to fate of reactive nitrogen and carbon. *Proc. Natl. Acad. Sci.* **118**, e2115204118 (2021).
12. LE Mitchell, et al., Observing and modeling the influence of layering on bubble trapping in polar firn. *J. Geophys. Res. Atmospheres* **120**, 2558–2574 (2015).
13. RH Rhodes, et al., Continuous methane measurements from a late Holocene Greenland ice core: Atmospheric and in-situ signals. *Earth Planet. Sci. Lett.* **368**, 9–19 (2013).
14. X Lan, KW Thoning, EJ lugokencky, Trends in globally-averaged CH4, N2O, and SF6 determined from NOAA Global Monitoring Laboratory measurements (2025).
